# Research on Processability and Transmission Performance of Low Temperature Co-Fired Ceramic Ball Grid Array Packaging Based on Electroless Plating Surface Modification for Microwave Transceiver Circuits

**DOI:** 10.3390/ma16206720

**Published:** 2023-10-17

**Authors:** Song Wang, Tianyu Hou, Rui Huo, Zhengtian Chen, Qinghua Zeng, Ying He, Yan Zhao, Xiao Liu

**Affiliations:** 1 School of Electronic Science and Engineering, University of Electronic Science and Technology of China, Chengdu 611731, China; wangsong@aircas.ac.cn; 2 Aerospace Information Research Institute, Chinese Academy of Sciences, Beijing 100094, China; houty@aircas.ac.cn (T.H.); huorui@aircas.ac.cn (R.H.); chenzt@aircas.ac.cn (Z.C.); zengqh@aircas.ac.cn (Q.Z.); zhaoyan@aircas.ac.cn (Y.Z.); liuxiao@aircas.ac.cn (X.L.)

**Keywords:** LTCC, ENEPIG, BGA packaging, T/R module, transmission performance

## Abstract

A microwave transmitter/receiver using the low-temperature co-fired ceramic substrate and ball grid array packaging demonstrates superior properties, including high integration, miniaturization, and high electromagnetic shielding. However, it holds limitations of inadequate hermeticity (that is, gas or moist impermeability), high cost, and low reproducibility. In this work, we aim to overcome these difficulties by introducing a new packing technique. The packaging utilizes an electroless plated Ni/Pd/Au surface, resulting in a significant enhancement of the packaging hermeticity by orders of magnitude, approaching the level of <5 × 10^−9^ Pa·m^3^/s. Both Sn63Pb37 and Au80Sn20 solder alloys demonstrate exceptional solderability, attributed to Pd atoms diffusing to the Au layer during soldering at 310 °C. A reliability test of the packaging shows that the shear strength of the solder balls drops after thermal shocks but negligibly affects the hermeticity of the packaging. Furthermore, a meticulously designed internal vertical interconnect structure and I/O interconnections were engineered in the ball grid array packaging, showcasing excellent transmission characteristics within the 10–40 GHz frequency range while ensuring effective isolation between ports.

## 1. Introduction

The traditional brick-shaped microwave transmitter and receiver (T/R) module utilizes soldering to establish interconnections between substrates, monolithic microwave integrated circuit (MMIC), connectors, and the metal box. The internal circuit interconnection is achieved through die bonding and wire bonding, and a hermetic package is formed by sealing the lid and the metal box. The brick-shaped package has found extensive application in high-reliability fields such as aerospace owing to its mechanical strength, heat dissipation capacity, reliability, maintainability, and testability [1]. However, due to constraints such as machining and connector size, achieving miniaturization poses a challenge [2,3]. An active electronically scanned array (AESA) consists of an active transmit and receive front-end for each antenna element. To achieve optimal scanning performance, the spacing between elements should be half a wavelength (λ/2). This also means that the transceiver circuits for each antenna unit need to be packaged within a square area with a side length of λ/2 [4]. This packaging requirement poses a bottleneck for the development of high-frequency AESA systems. Additionally, the brick-shaped packaging of transceiver components brings about difficulties in cost reduction, weight reduction, and production efficiency improvement [5]. Therefore, as the demand for low-cost, lightweight, and compact phased array systems in millimeter wave increases, the brick-shaped package struggles to accommodate smaller unit spacing and low antenna profile sizes.

Multi-chip device packaging has emerged as a prominent research area for T/R modules in response to the growing need for miniaturization [6]. Silicon-based system-on-chip (SoC) technology, along with packaging techniques utilizing interposers, has garnered significant attention in the field of high-speed communication [7,8]. These advancements have facilitated the realization of micrometer-scale lines and vias capable of operating within the millimeter-wave to sub-terahertz frequency range. Building upon the concept of system-level packaging, further miniaturization of phased array antennas can be achieved by integrating more circuits, such as radiation front, need networks, and beamforming, with the transceiver circuitry [9,10].

However, for high-reliability, high-power, and small-batch applications, such as aerospace, these approaches face practical challenges due to high development costs and difficulties. In contrast, ceramic-based ball grid array (BGA) packaging technology offers exceptional reliability, excellent heat dissipation capability, and relatively high integration and miniaturization potential, making it a more attractive option [11]. Using a three-dimensional interconnect substrate, it is possible to flexibly convert between coaxial, strip-line, and microstrip-line structures [12]. This allows for relocating package interfaces to the bottom of the package, reducing the package footprint while minimizing transmission loss, and ensuring sufficient channel isolation [13,14]. In terms of I/O structure, its coaxial distributed solder ball structure exhibits excellent shielding capabilities for transmitting high-frequency signals [15,16]. Low temperature co-fired ceramic (LTCC) substrates are widely employed in the production of microwave circuit boards and devices [17]. This is due to their exceptional ability for three-dimensional wiring, low dielectric constant, and minimal dielectric loss [18]. With advantages such as virtually unlimited layering capability, flexible via interconnections, and cost-effective cavity fabrication, LTCC substrates find extensive applications in miniaturized microwave components and substrates [19]. 

This paper investigates the application of ball grid array packaging, with LTCC as the package substrate and Sn63Pb37 soldering balls as the electrical interconnection terminals. To ensure a hermetic package, a Kovar window frame is soldered onto the substrate and sealed with a lid using parallel sealing. The utilization of LTCC, known for its remarkable mechanical strength and chemical stability, has significantly reduced the reliance on metal structures. By employing BGA solder balls to create input/output ports for RF and low-frequency signals, the need for connectors is eliminated. This breakthrough leads to a substantial increase in interconnect density and a remarkable reduction in package size.

However, there are certain limitations associated with the use of LTCC substrates in BGA packaging. First, the lower sintering temperature of LTCC, in comparison with traditional ceramic substrates such as high temperature co-fired ceramic (HTCC), leads to a less dense microstructure and lower mechanical strength [20]. Consequently, for high-reliability applications when considering LTCC as a packaging substrate, its reliability needs to be carefully assessed [21]. Specifically, as one of the crucial factors affecting long-term stability, research on the hermeticity of LTCC exhibits significant discrepancies, ranging from 10^−6^ Pa/(m^3^·s) to 10^−11^ Pa/(m^3^·s) [22,23]. On the other hand, HTCC has gained widespread recognition for its hermeticity, which can achieve levels ranging from 10^−9^ Pa/(m^3^·s) to 10^−10^ Pa/(m^3^·s) [17,24]. Second, reducing the cost of conductor pastes that employ gold and silver systems proves to be challenging, thus restricting its application in low-cost packaging [17]. Third, due to the diverse requirements of post-assembly processes, the formulation of co-fired thick film conductors tends to be complex, resulting in intricate procedures and difficulties in improving yield [18]. Hence, it is essential to investigate the mechanisms that limit the hermeticity of LTCC substrates and implement necessary improvements while simplifying the process and reducing costs. Additionally, when considering the transmission structure of BGA packaged T/R modules, it becomes imperative to apply transmission line theory to ensure optimal high-frequency characteristics such as characteristic impedance, insertion loss, isolation, and more.

Here, we address these challenges by focusing on the utilization of silver-based conductors and surface modification through the electroless plated Ni/Pd/Au (ENEPIG) process. Preliminary verification has been conducted for its application in BGA [25] and LGA [26] packaging on copper-clad substrates. The reasons behind the insufficient hermeticity of traditional LTCC fabrication processes are analyzed, and the hermeticity of thick film conductors with ENEPIG surface modification, as well as the wetting ability with Sn63Pb37 and An80Sn20 solders, are studied. The reliability of BGA packaging using this improved process is also investigated.

The distributed parameter characteristics of microwave circuit transmission structures are heavily dependent on the properties of materials (both dielectric and conductive) and manufacturing capabilities. Careful design is required for the transmission structures within the substrate [13] and the interconnection interfaces with the motherboard [14,27]. To validate the feasibility of utilizing LTCC-BGA packaging with Ag-based material for ENEPIG surface modification as a conductive medium, a three-dimensional electromagnetic field analysis is conducted to design and simulate the internal vertical interconnection structure within LTCC and BGA transmission interfaces. This is carried out to assess its suitability for applications within the 10–40 GHz frequency range.

## 2. Experimental and Simulation Methods

### 2.1. LTCC Substrate Preparation

**Figure 1:**
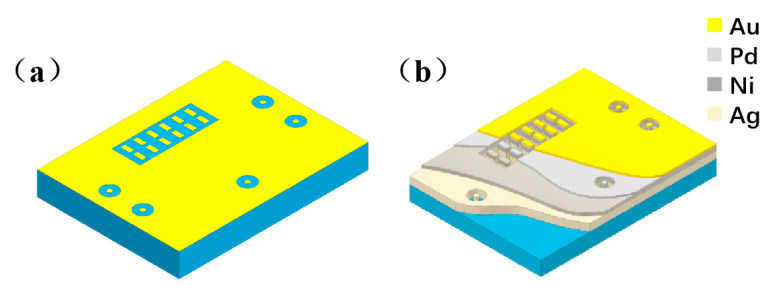
LTCC substrate sample preparation. (**a**) Schematic representation of the substrate outline; (**b**) schematic structure of the surface metal layer.

The multi-layer co-fired thick film conductors in LTCC substrates are produced using a single silver-based paste, with the dielectric material chosen as the FST07 (Guangdong Fenghua Special Electronic Conponents Co., Ltd., Zhaoqing, China) glass–ceramic composite system, which has a dielectric constant of 6.6 and a dielectric loss tangent value of less than 0.007. An electroless plating process is employed to deposit Ni, Pd, and Au layers on the surface of the silver conductor on the substrate. The thickness measurements of the Ag, Ni, Pd, and Au coatings are determined to be 8–10 μm, 4–5 μm, 0.15–0.18 μm, and 0.12–0.15 μm, respectively, utilizing the X-ray fluorescence spectrometric method. The substrate and its coated structure are illustrated in Figure 1. The three-dimensional circuit network includes surface solder pads, vias, and inner layer interconnections. The surface multi-layer metal layers, from inside to outside, consist of Ag, Ni, Pd, and Au.

### 2.2. Hermeticity Test

The following methods are employed for the hermeticity test: fine leak detection and rough leak detection. In fine leak detection, the samples are pressurized with 100% helium tracer gas at 414 kPa for 2 h within a sealed chamber. Subsequently, the samples are transferred to a vacuum chamber connected to a helium mass spectrometer leak detector to measure the release of helium from the samples and determine the leakage rate. For rough leak detection, the samples are immersed in a solution of trichlorotrifluoroethane and maintained at 620 kPa for 3 h. Then, the samples are placed in a solution of perfluorotributylamine at 125 °C, and careful observation is made to identify the emergence of bubbles and their respective locations.

### 2.3. Solderability Test

**Figure 2:**
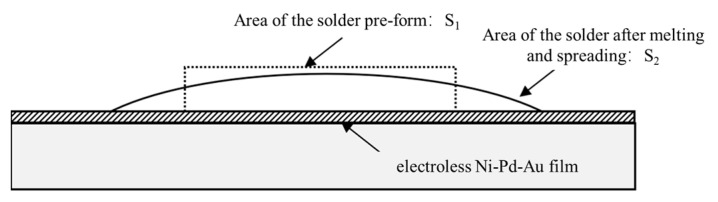
Expansion rate solderability test.

The expansion rate method, based on the American Society for Testing and Materials standard ASTM-B-545 [28], is employed to assess the wettability of Sn63Pb37 and Au80Sn20 solder on ENEPIG. The schematic diagram of this method is illustrated in Figure 2.

To start, a solder pre-form is positioned onto the ENEPIG pad. Subsequently, the temperature of the substrate is elevated until it reaches 30 °C above the solder’s melting point, allowing the solder to wet and distribute evenly across the substrate. Finally, the expansion rate is calculated using example Equation (1), which defines it as the ratio of the change in the solder area after melting to the original area.

(1) $$E=\frac{S_{2}-S_{1}}{S_{1}}\times 100\%,$$

where *E* is the expansion rate, *S*_1_ is the area of the solder pre-form before soldering (mm^2^), and *S*_2_ is the area of the solder pre-form after melting and spreading (mm^2^).

### 2.4. Reliability Test

**Figure 3:**
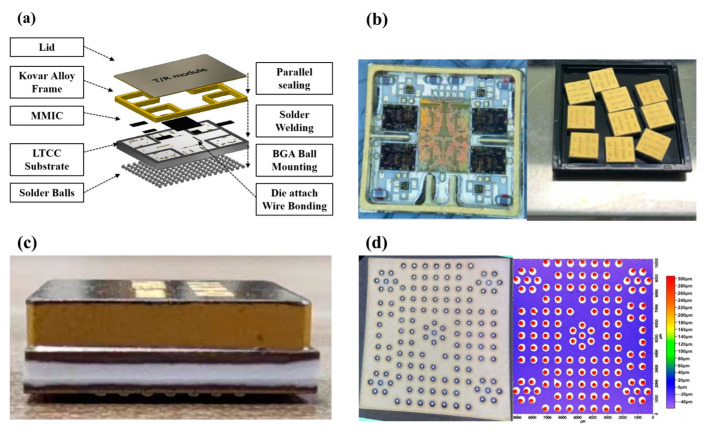
BGA packaged T/R modules. (**a**) Schematic of LTCC-BGA packaging; (**b**) internal circuitry and sample preparation; (**c**) side view; (**d**) backside solder balls and height consistency.

To evaluate the long-term reliability of the BGA packaging, a batch of samples is fabricated following the packaging architecture depicted in Figure 3a. An LTCC substrate with an array of pads (0.3 mm in diameter) is fabricated, and the surface silver pads are modified using the ENEPIG process. Subsequently, Sn63Pb37 solder balls (0.4 mm in diameter) are placed and reflowed under a nitrogen atmosphere. The Kovar frame is brazed onto the LTCC substrate using An80Sn20 solder (Guangzhou Xianyi Electronic Technology Co., Ltd., Guangzhou, China) and sealed in parallel with the lid. The internal circuitry and the sample preparation of the T/R component are illustrated in Figure 3b, while the side view and bottom morphology are shown in Figure 3c,d. Laser profilometer is employed to perform a surface scan for the solder ball heights, with a difference of less than 10 μm.

The test conditions for reliability can be found in Table 1. Before and after conducting the reliability tests, measurements are taken to determine the fine leak detection results (as mentioned in Section 2.2) and the shear force of the solder joints. In the ball shear test, a testing probe is used to apply a parallel force to the solder ball in the direction aligned with the solder pad. The shear height is set to 100 µm, and the test speed is maintained at 100 µm/s until the solder ball experiences destructive failure. A sensor records the force applied by the testing probe over time, and the maximum value is considered as the shear force for that particular solder ball.

### 2.5. Characterization of Morphology and Composition

The thickness of the coatings is measured using an X-ray fluorescence thickness gauge (Thick800A, Jiangsu Skyray Instrument Co., Ltd., Kunshan, China). In order to obtain a comprehensive understanding, the morphology and composition of the samples are meticulously characterized. The surface morphology is visualized by employing scanning electron microscopy (SEM) with SU5000 (Hitachi Ltd., Tokyo, Japan) to examine the microstructure and surface features. Elemental composition analysis is conducted through energy-dispersive X-ray spectroscopy (EDX) with Ultim Max (Oxford Instruments plc., Oxford, UK) to identify the elements present in the samples. Moreover, the surface chemistry of the LTCC substrates after the Au80Sn20 soldering process is studied using X-ray photoelectron spectroscopy (XPS) analysis, utilizing the Thermo Scientific Escalab 250Xi Photoelectron Spectrometer (Thermo Fisher Scientific Inc., Uppsala, Sweden). The post-solder ball height of the BGA is characterized using a CT 300 laser profilometer (Cyber Technologies, Düsseldorf, Germany).

### 2.6. Computational Modeling

**Figure 4:**
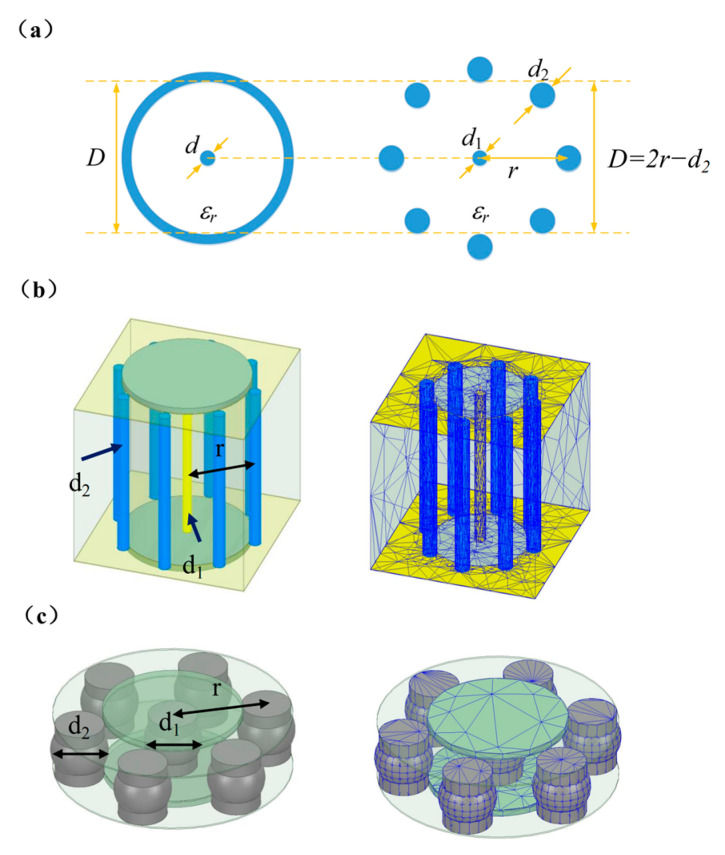
The constructed (**a**) coaxial and quasi-coaxial structures corresponding to (**b**) the vertical interconnection structure inside LTCC and (**c**) the BGA interconnection structure.

The coaxial and quasi-coaxial structures are illustrated in Figure 4a. On the left side of the diagram is the coaxial structure, comprising a central conductor (often copper wire) surrounded by a metal shield layer, with insulation separating the two. This structure effectively reduces signal interference and loss, providing lower transmission losses and higher signal quality. However, implementing coaxial structures in multi-layer circuit boards can be challenging. To achieve similar high-frequency signal transmission characteristics, the quasi-coaxial structure is frequently employed in the design of multi-layer circuit boards. It consists of a central via hole surrounded by several discrete shield vias. HFSS software (R16 version) is utilized for the analysis of microwave transmission properties in the quasi-coaxial vertical interconnection structure as well as its grid segmentation in LTCC (Figure 4b) and the BGA structure (Figure 4c).

In Figure 4b, the blue cylinders surrounding the ground holes, with a diameter of d_2_, can be considered as the outer conductor shielding layer of coaxial connectors. Meanwhile, the yellow cylinder at the center, with a diameter of d_1_, represents the inner conductor. The through-hole filling material is configured as Ag. In a similar manner, the placement of BGA solder balls can follow a similar approach. As typically only one diameter of BGA solder balls is used for packaging, in the quasi-coaxial structure formed by the BGA balls, let us denote the diameter as d, where d_1_ = d_2_. The material for the BGA balls is set as Sn63Pb37, while the upper and lower solder pads are made of Ag. As the number of ground holes “N” approaches infinity, the quasi-coaxial vertical interconnection structure transforms into a standard coaxial line.

## 3. Results and Discussion

### 3.1. Hermeticity Test of the Packaging

**Figure 5:**
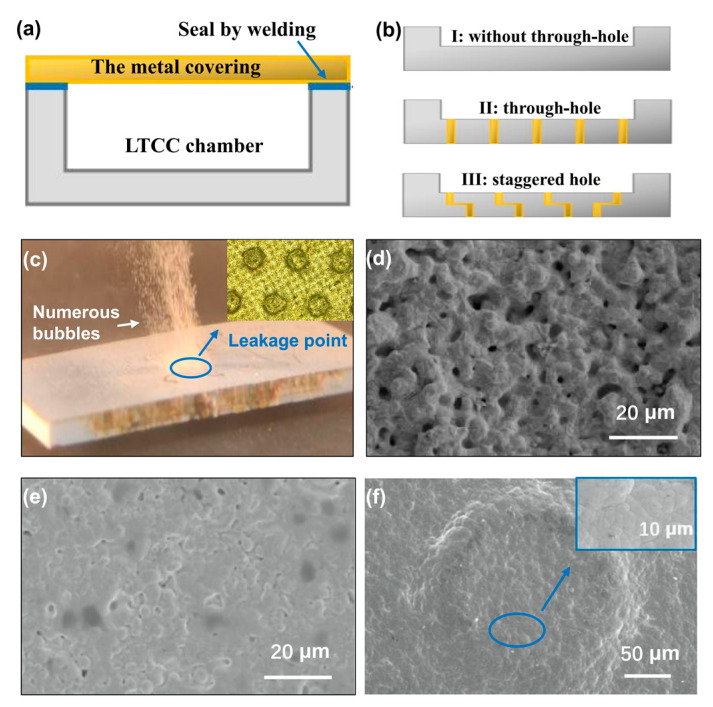
The results of the leakage rate tests and SEM images of samples: (**a**–**c**) without modification by ENEPIG; (**d**–**f**) with modification by ENEPIG.

The samples depicted in Figure 5a are specifically created to conduct a hermeticity test on LTCC substrates fabricated through conventional methods, where the lid and LTCC chamber are sealed using tin–lead soldering. Taking into consideration the practical LTCC packaging design, in order to ensure microwave device grounding and heat dissipation, it is common to create through-hole grounding at the bottom of the cavity. To investigate the impact of different through-hole designs on packaging hermeticity, three types of through-hole configurations are designed as shown in Figure 5b: without through-hole, through-hole, and staggered-hole.

The rough leak detection results of the three samples indicate that, except for the sample with through-holes showing evident leakage points, the other two samples pass the testing successfully. Figure 5c illustrates the leakage condition during the rough leak testing of the sample with through-holes, along with the position of the through-holes. It is evident that the region of bubble overflow is concentrated around the through-hole located at the bottom of the cavity. Upon examining the microstructure of the through-hole area (Figure 5d), it becomes apparent that lower sintering temperatures result in a decreased density of the through-hole conductor paste, leading to the presence of numerous voids measuring 1 to 3 μm. This poses a potential risk of leakage in a hermetic package. By optimizing the filling process, an improvement in the density of the through-hole can be achieved. However, there remains a significant likelihood that it may fail the rough leak detection. Conversely, due to the bridging effect of the glass phase during the sintering process, the ceramic material (Figure 5e) attains a sufficiently high density, eliminating channels for gas leakage.

While adopting the approach of not having through-holes or using staggered holes may potentially meet the hermeticity requirements of LTCC packaging, it is not conducive to power device heat dissipation and the low impedance grounding requirements of microwave circuits for signal paths. Figure 5f illustrates the through-hole area after being coated with ENEPIG. It is evident that the originally porous surface undergoes a significant transformation into a highly compact morphology. Consequently, during the rough leak detection (Figure 5f), there is no further occurrence of bubble overflow. In addition, the fine leak detection test confirms a hermeticity lower than 5 × 10^−9^ Pa·m^3^/s, which closely approaches the HTCC standard.

### 3.2. Solderability

**Figure 6:**
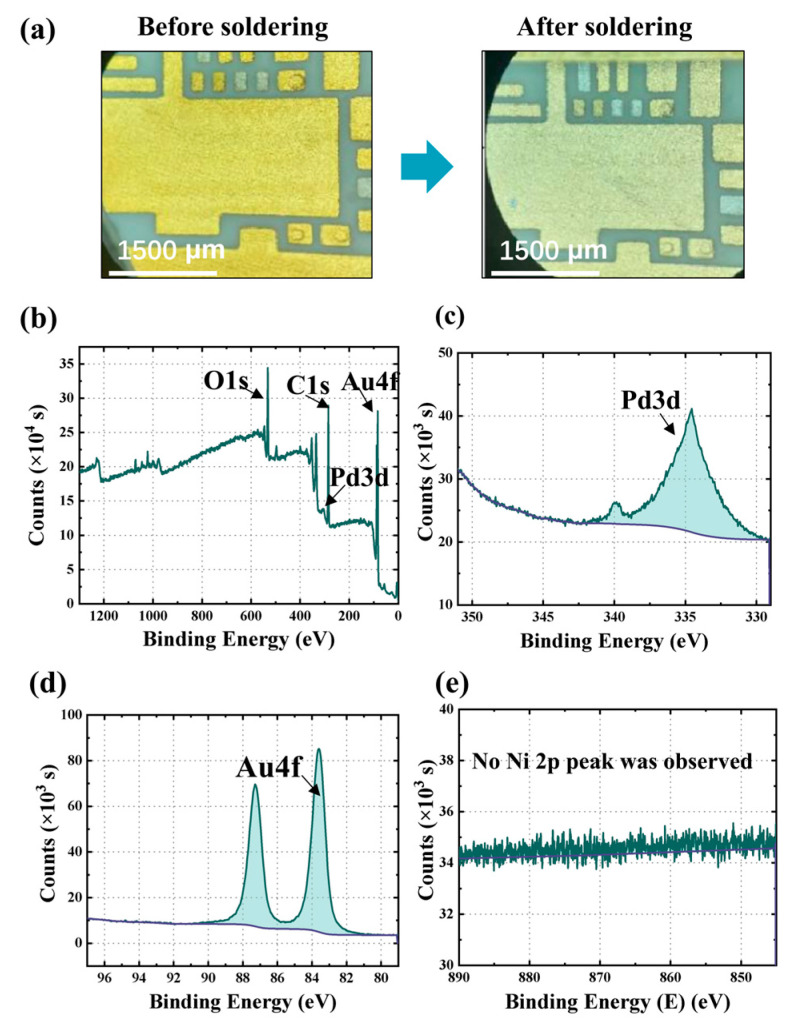
Surface element analysis: (**a**) The color change of ENEPIG coating before and after Au80Sn20 soldering; (**b**) long-range energy spectrum diagram; (**c**) Pd 3d fine spectrogram; (**d**) Au 4f fine spectrogram; (**e**) Ni 2p fine spectrogram.

The expansion rates of Sn63Pb37 and Au80Sn20 solder alloys are determined by calculating the area ratio after wetting and spreading on the substrate surface using Equation (1). The results show that the expansion rates for Sn63Pb37 and Au80Sn20 are 94% and 88%, respectively, indicating excellent solderability of the pads after ENEPIG plating. It is worth noting that, upon exposure to the soldering temperature of 310 °C, the color of the ENEPIG coating noticeably lightens during the soldering process with Au80Sn20 (as shown in Figure 6a), which is not observed in the wetting test performed with Sn63Pb37.

To investigate the temperature-dependent diffusion of the metal film, surface composition analysis is conducted using X-ray photoelectron spectroscopy (XPS) analysis, as shown in Figure 6b–e. The long-range energy spectrum (Figure 6b) reveals the presence of elements, including O, C, Pd, and Au, on the surface, along with additional peaks attributed to surface adsorbents. Refined energy spectra of the Pd 3d and Au 4f photoelectron peaks after substrate correction are presented in Figure 6c,d. The elemental sensitivity factor method is utilized to calculate the photoelectron peak areas of Pd and Au, resulting in a surface Pd-to-Au atom ratio of approximately 1:5. This observation indicates that, at 310 °C, Pd atoms diffuse towards the surface of the Au layer, resulting in a color change. It is worth noting that the remarkable antioxidant capability and high surface energy of Pd [21] contribute to the preservation of surface wettability and bondability. Conversely, Figure 6e illustrates the absence of an evident Ni 2p peak within the 845–890 eV range, suggesting the absence of Ni elements on the surface. This finding confirms that the dense Pd layer acts as a diffusion barrier, preventing Ni atoms from reaching the surface. While the Ni layer demonstrates favorable solderability, it easily oxidizes in ambient air, which consequently diminishes its wetting ability with solder.

**Figure 7:**
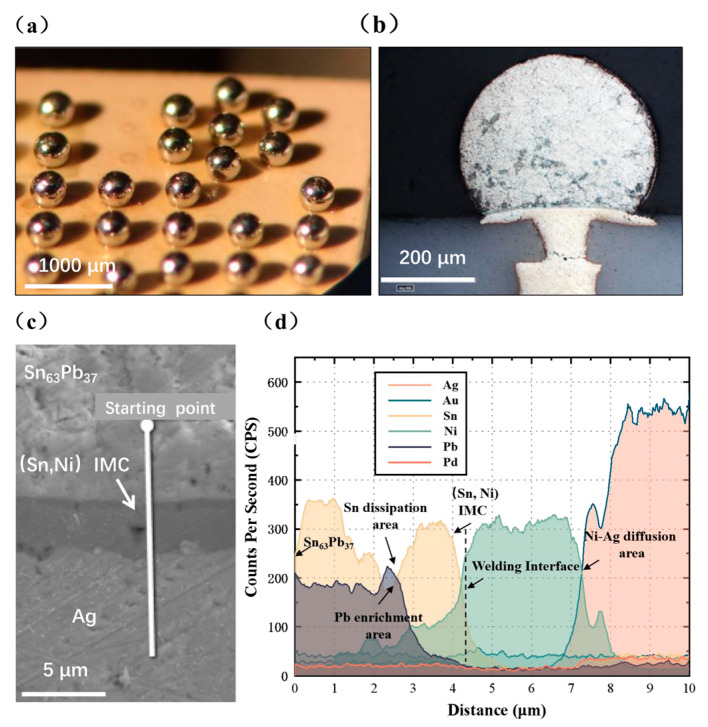
Analysis of the solder joint interface of Sn63Pb37 solder balls: (**a**) side view of solder balls; (**b**) cross-sectional metallographic; (**c**) EDS line-scanning; (**d**) elemental variation at the interface.

Figure 7 exhibits the morphology photo, cross-sectional metallographic image, and EDS line-scanning outcomes, capturing the interface between solder balls and enhanced LTCC substrates. As illustrated in Figure 7a,b, the solder ball surface appears luminous and exhibits a spherical shape and demonstrates excellent wetting with the pads, devoid of any pores or voids. Figure 7c,d depicts the distributions of Ag, Ni, Pd, Au, Sn, and Pb elements. Notably, the Au and Pd contents remain consistently low and exhibit no variation across the entire scanning range. This observation suggests that, during the soldering process, the surface layer of Au and Pd swiftly diffuses into the Sn63Pb37 solder without aggregating and forming intermetallic compounds at the interface.

Initially, the analysis of atomic percentages reveals that the mass percentage of Sn is approximately 65%, closely resembling the composition ratio of Sn63Pb37. Progressing along the scanning direction, the Sn content gradually decreases, while the Pb content increases. This phenomenon arises from the differential diffusion rates between Sn and Pb. Sn atoms exhibit a faster diffusion rate towards the soldering interface. Consequently, they react with Ni, leading to the formation of various intermetallic compounds (IMCs) such as Ni_3_Sn_4_, Ni_3_Sn_2_, Ni_3_Sn, etc. [25,29,30]. This reaction consumes some Sn atoms in adjacent regions, consequently impeding the diffusion of Pb. Consequently, this process results in the enrichment of Pb atoms near the IMC, signifying the resistance to Pb diffusion.

The distribution of Ni exhibits significant two-way diffusion towards both the solder and the underlying Ag conductor. The diffusion distance is approximately 3.5 µm towards the solder and 2 µm towards the Ag conductor. This indicates that the Ni layer, acting as an adhesive, forms a close bond with both the solder and the underlying thick film conductor during soldering. Consequently, this bonding mechanism greatly enhances the strength of the solder joint, improving its overall structural integrity.

### 3.3. Reliability Test

**Figure 8:**
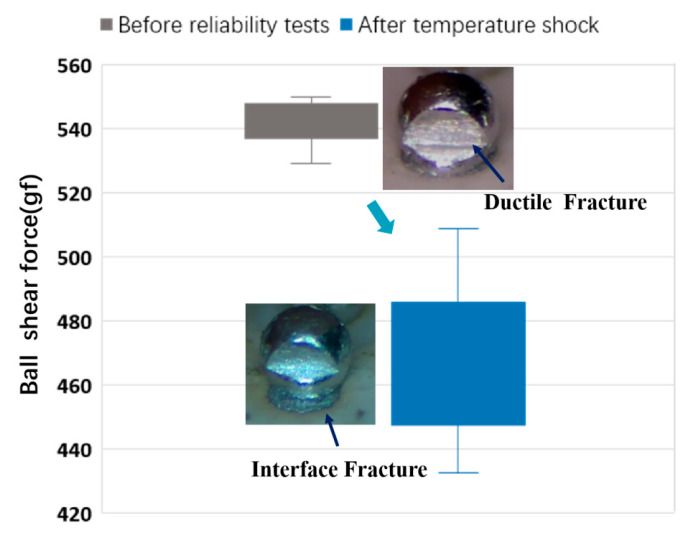
Shear force of solder balls before and after temperature shock testing.

The shearing test results before and after temperature shock are presented in Table 2 and Figure 8. The average shear force, based on twenty measurements, before and after the temperature shock experiment, exceeds 400 gf. Subsequently, the average shear force of the solder ball decreases by 79.7 gf after the temperature shock experiment compared with the pre-experiment value, accompanied by a one-order increase in the standard deviation.

Before the temperature shock tests, a ductile fracture is observed within the solder balls, indicating significant plastic deformation during the shearing test but remaining attached to the solder pad. However, the temperature shock tests reveal a different shear failure mechanism. The solder balls undergo plastic deformation, and ultimately, when deformation reaches a critical point, the bottom of the solder ball detaches from the pad, resulting in an interface fracture at the interfacial IMC. This suggests the need for precise control over IMC thickness during the ball-planting process to ensure strong soldering joints between BGA balls and LTCC pads.

The analysis demonstrates that, at the high-temperature stage (150 °C) of the thermal shock tests, Sn diffusion accelerates. This leads to continuous growth and thickening of the intermetallic compound (IMC) layer at the connection interface [25,30]. Consequently, the solder joint’s strength is significantly weakened, causing a decrease in bonding strength. These findings highlight the critical role of controlling IMC thickness in the ball-planting process to ensure strong soldering joints between BGA balls and LTCC pads.

**Figure 9:**
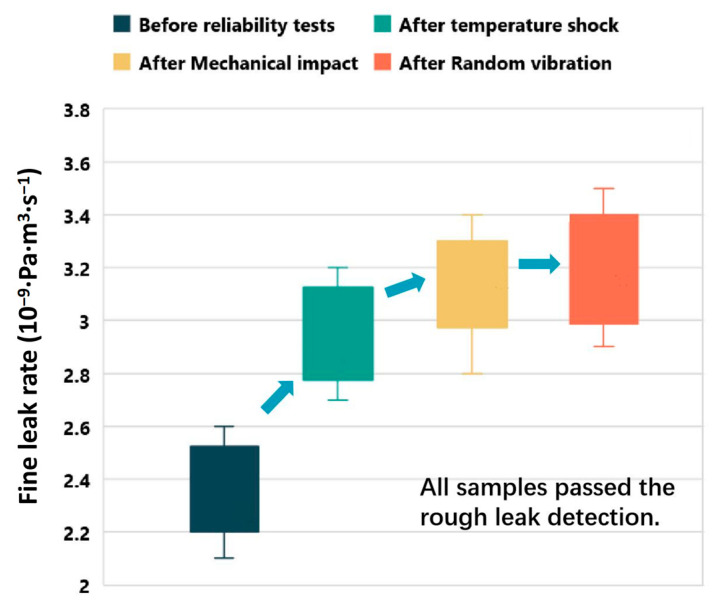
Hermeticity assessment of simulated BGA-packaged T/R modules pre- and post-reliability testing.

The hermeticity of the simulated BGA-packaged T/R modules is evaluated before and after conducting reliability tests, and the findings are presented in Table 3 and Figure 9. All samples pass the initial rough leak detection, and although the leakage rate of fine leak detection slightly increases with the temperature shock test, it remains below 5 × 10^−9^ Pa·m^3^/s. The impact of mechanical shock and random vibration on the results of the fine leak test can be considered negligible. This signifies that BGA packaging utilizing ENEPIG LTCC offers a consistent and stable working environment for the internal circuits of the components, ensuring long-term functionality.

### 3.4. Computation Results and Discussion

#### 3.4.1. Qual-Coaxial Vertical Interconnection Structure in LTCC Substrates

The cutoff frequency of qual coaxial vertical interconnection structures (as shown in Figure 4) is related to the distance r between the axis of the signal hole and the ground hole, as shown in Equation (2):

(2) $$r=\frac{c}{f_{c}\pi *\sqrt{\varepsilon_{r}}}+\frac{d_{2}-d_{1}}{2},$$

Table 4 illustrates the computed values of fc at different *r* values in an LTCC substrate with a dielectric constant of 6.6. It is evident that, as *r* decreases, the corresponding *fc* value increases. Nevertheless, it can be difficult to achieve excessively small inter-hole distances due to limitations associated with substrate manufacturing processes. In such cases, one possible solution is to select a material with a lower dielectric constant to strike a balance between processability and feasibility.

To ensure the absence of higher-order modes within the operating frequency range, which does not exceed 40 GHz, it is imperative to limit the value of *r* to ≤0.95 mm. Taking into account a certain margin for the frequency range in practical applications and aiming to provide as many solder balls as possible (i.e., reducing *r*) to enhance the assembly strength while considering process feasibility, we select a distance of *r* = 0.65 mm. To ensure manufacturability and yield of the LTCC substrate, as well as to reduce the size of the transmission structure, we select pore sizes for the signal hole (*d*_1_ = 0.127 mm) and ground holes (*d*_2_ = 0.17 mm), along with a total of eight ground holes (N = 8), thereby calculating the characteristic impedance Z_0_ to be 51.05 Ω.

**Figure 10:**
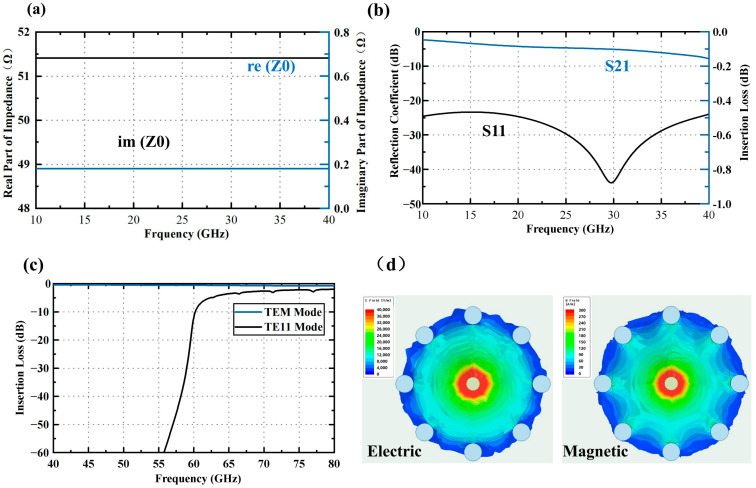
Curve of impedance, reflection coefficient, and insertion loss with frequency of the qual-coaxial vertical interconnection structure in LTCC substrates: (**a**) impedance; (**b**) reflection coefficient (S_11_) and insertion loss (S_21_); (**c**) transmission loss of TEM and TE_11_; (**d**) distribution of electric and magnetic fields with quasi-coaxial structure.

The HFSS model simulation results of the qual-coaxial vertical interconnection structure in LTCC substrates are shown in Figure 10. Figure 10a,b indicate that, in the wide frequency band of 10~40 GHz, the characteristic impedance “im(Z0)” is 51.41 Ω, the reflection coefficient “S_11_” is less than −20 dB, and the transmission coefficient “S_21_” is less than 0.2 dB, indicating excellent transmission characteristics. Figure 10c displays the transmission losses of both TEM and TE_11_ modes in qual-coaxial structures. Notably, for frequencies below 60 GHz, the transmission loss remains below −10 dB, indicating the superior suppression effect of the qual-coaxial structure on the first higher-order mode, TE_11_. These simulation results align with the cutoff frequency calculated using Equation (2) (as shown in Table 4), further validating their accuracy. As illustrated in Figure 10d, the electromagnetic field distribution of the coaxial-like structure and the coaxial structure exhibit a noticeable contrast, demonstrating that both structures effectively confine the electromagnetic field from leaking outside in the propagation direction.

#### 3.4.2. Qual-Coaxial BGA Vertical Interconnection Structure Interconnected with External Circuits 

In Figure 4c, similar to the quasi-coaxial structure in Figure 4b, BGA solder balls can also be arranged in a quasi-coaxial configuration. We perform preliminary simulations to determine the characteristic impedances of BGA quasi-coaxial structures with varying solder ball diameters and pitches, as shown in Table 5. In the table, the symbol “×” indicates configurations that are not recommended according to the BGA packaging design specifications.

Achieving a characteristic impedance of 50 Ω in the quasi-coaxial BGA vertical interconnection structure poses challenges. However, considering the high-density interconnection demands of the BGA-packaged T/R module, we set the solder ball diameter and pitch in the model to 0.4 mm and 0.65 mm, respectively.

**Figure 11:**
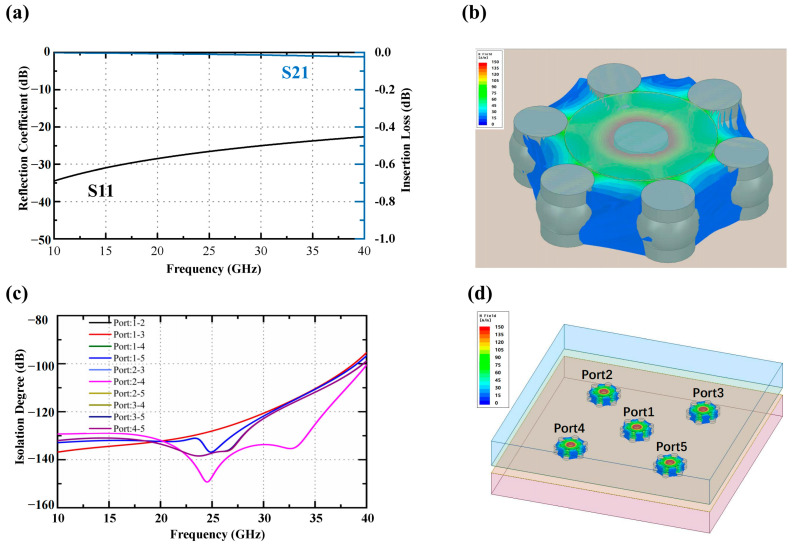
Simulated reflection coefficient and insertion loss, electromagnetic field distributions, and isolation of tile T/R module with quasi-coaxial port using BGA structure: (**a**) reflection coefficient (S_11_) and insertion loss (S_21_); (**b**) electromagnetic field distribution of a single-port configuration; (**c**) isolation between the ports; (**d**) electromagnetic field distribution of a multi-port configuration.

The simulation results of the qual-coaxial BGA interface structure interconnected with external circuits are presented in Figure 11a. The results demonstrate excellent transmission characteristics within the wide frequency range of 10 to 40 GHz, with a reflection coefficient “S_11_” below −20 dB and a transmission loss “S_21_” below 0.1 dB. Figure 11b,c showcase the electromagnetic field distribution map and the curve depicting the isolation degree’s frequency dependence for the quasi-coaxial BGA vertical interconnection structure. The electromagnetic field distribution reveals that the propagation direction effectively confines the leakage of the electromagnetic field. Furthermore, the isolation degree between different BGA qual-coaxial ports, spanning from 10 to 40 GHz, exceeds 90 dB. These findings signify that the structure meets the stringent isolation requirements of each port in the phased array antenna radiation unit as well as the input and output ports of the T/R module.

In summary, the qual-coaxial structures within the LTCC board and BGA solder balls exhibit comparable millimeter wave signal propagation performance to the conventional coaxial structure, making it suitable for application in the proposed BGA-packaged T/R module discussed in this article.

## 4. Conclusions

This study presents a ball grid array packaging solution that utilizes a low-temperature co-fired ceramic substrate to meet the growing need for miniaturization and integration of T/R modules. The inclusion of surface electroless plating layers, consisting of Ni/Pd/Au films, significantly enhances reliability while reducing costs. These desirable features make this approach valuable in addressing current challenges. This research assesses the processability, reliability, and transmission characteristics of the proposed packaging structure, demonstrating its high application value for radar and communication products utilized in high-reliability applications. The findings indicate that:

(1) The poor density of the filled paste at the through-hole is the fundamental reason for the inadequate hermeticity of traditional LTCC substrates. By using a surface-plated dense Ni/Pd/Au film layer, the hermeticity of LTCC packaging can be effectively improved to a level of 10^−9^ Pa·m^3^/s.
(2) The Sn63Pb37 and Au80Sn20 solder alloys exhibit excellent solderability. The mechanism study shows that, under high-temperature soldering at 310 °C, Pd atoms diffuse to the surface of the Au layer, improving the bondability of the solder and the substrate but not noticeably affecting solderability.
(3) In the thermal cycling test, the growth of intermetallic compounds at the soldering interface is facilitated, resulting in a slight decrease in the average shear strength of the solder balls and an increase in the dispersion of numerical distribution. On the other hand, hermeticity of the packaging remains <5 × 10^−9^ Pa·m^3^/s after the reliability test.
(4) The interconnection structures fabricated using the aforementioned process are studied and optimized. The internal vertical interconnect structure and I/O interconnections of the BGA packaging substrate are designed, exhibiting excellent transmission characteristics within a wide frequency range of 10–40 GHz and good isolation between ports.

Further efforts are required in the areas of board-level packaging applications and thermal management. Building upon this study, our future endeavors aim to integrate active circuits and develop a multi-channel integrated transceiver module with comprehensive receiving and transmitting capabilities. This module will be combined with board-level assembly to validate its electrical performance and reliability. Furthermore, an assessment of its heat dissipation capability will also be conducted.

## Figures and Tables

**Table 1:** Reliability verification test conditions.

Environmental Testing Methods	Condition
Temperature shock	Temperature range: −65~150 °C, maintain for 15 min at −65 °C and 150 °C, 50 cycles
Mechanical impact	Peak accelerations 1500 g, peak pulse width 0.5 ms
Random vibration	Power spectral density 30 (m/s^2^)^2^/Hz, total root mean square of acceleration 207.1 m/s^2^

**Table 2:** Results of shearing tests.

Testing Results	Before Temperature Shock	After Temperature Shock
Maximum shear force (gf)	549.8	508.7
Minimum shear force (gf)	529	432.5
Average shear force (gf)	542.4	467.2
Standard deviation (gf)	6.1	22.1

**Table 3:** Results of fine leak detection.

No.	Fine Leak Detection (×10^−9^ Pa·m^3^·s^−1^)			
	Before Temperature Shock	After Temperature Shock	After Mechanical Impact	After Random Vibration
1	2.5	3.2	3.2	3.2
2	2.2	3.0	3.2	3.2
3	2.3	2.7	2.8	2.9
4	2.1	2.9	4.1	3.3
5	2.6	3.2	3.4	3.5
6	2.3	3.1	3.3	3.2
7	2.2	2.7	2.9	2.95
8	2.6	2.9	3.2	3.4
9	2.4	2.8	3.0	3
10	2.3	2.9	3.3	3.4

**Table 4:** The relationship between cutoff frequency and spacing *r* of vertical interconnection quasi-coaxial structure of LTCC.

*f_c_* (GHz)	*r* (mm)	*f_c_* (GHz)	*r* (mm)
30	1.30	70	0.55
40	0.95	80	0.48
50	0.76	90	0.43
60	0.64	100	0.39

**Table 5:** Typical characteristic impedance of radio frequency BGA structure.

	*r* = 0.65 mm	*r* = 0.70 mm	*r* = 0.75 mm	*r* = 0.80 mm
*d* = 0.30 mm	84.80	90.33	95.37	100.01
*d* = 0.40 mm	62.44	68.37	73.77	78.75
*d* = 0.45 mm	×	×	64.49	69.63
*d* = 0.50 mm	×	×	×	61.15

## Data Availability

The data that support the findings of this study are available from the corresponding author, upon reasonable request.
